# Antimalarial Activity and Toxicological Assessment of *Betula alnoides* Extract against *Plasmodium berghei* Infections in Mice

**DOI:** 10.1155/2019/2324679

**Published:** 2019-11-13

**Authors:** Prapaporn Chaniad, Tachpon Techarang, Arisara Phuwajaroanpong, Chuchard Punsawad

**Affiliations:** ^1^School of Medicine, Walailak University, Nakhon Si Thammarat 80160, Thailand; ^2^Tropical Medicine Research Unit, Research Institute for Health Sciences, Walailak University, Nakhon Si Thammarat 80160, Thailand

## Abstract

The resistance of malaria parasites to the current antimalarial drugs has led to the search for novel effective drugs. *Betula alnoides* has been traditionally used for the treatment of malaria, but the scientific evidence to substantiate this claim is still lacking. Therefore, the present study aimed at evaluating the antimalarial activity and toxicity of an aqueous stem extract of *B. alnoides* in a mouse model. The *in vivo* antimalarial activity of an aqueous stem extract of *B. alnoides* was determined by a 4-day suppressive test in mice infected with chloroquine-sensitive *Plasmodium berghei* ANKA. The *B. alnoides* extract was administered orally at different doses of 200, 400, and 600 mg/kg body weight. The levels of parasitaemia, survival time, body weight change, and food and water consumption of the mice were determined. The acute toxicity of the extract was assessed in the mice for 14 days after the administration of a single oral dose of 5000 mg/kg. An aqueous stem extract of *B. alnoides* exhibited a significant dose-dependent reduction of parasitaemia in *P. berghei*-infected mice at all dose levels compared to the reduction in the negative control. Extract doses of 200, 400, and 600 mg/kg body weight suppressed the levels of parasitaemia by 46.90, 58.39, and 71.26%, respectively. The extract also significantly prolonged the survival times of the *P. berghei*-infected mice compared to the survival times of the negative control mice. In addition, at all dose levels, the extract prevented body weight loss in *P. berghei*-infected mice. For the acute toxicity, there were no significant alterations in the biochemical parameters and in the histopathology. In conclusion, the aqueous stem extract of *B. alnoides* possesses antimalarial properties. A single oral dose of 5000 mg/kg body weight had no significant toxic effects on the function and structure of the kidneys and liver. These results support its use in traditional medicine for the treatment of malaria.

## 1. Introduction

Malaria is a major parasitic disease that occurs in tropical and subtropical regions and is caused by intraerythrocytic protozoa of the *Plasmodium* genus [1]. Among the five species of human malaria parasites, *Plasmodium falciparum* is responsible for the most severe type of human malaria and has developed resistance to the existing antimalarial drugs [2]. In 2017, there were an estimated 219 million malaria cases worldwide with a mortality of 435,000 cases [3]. The emergence and rapidly developing multidrug-resistant strains of *Plasmodium*, particularly *P. falciparum*, represent a critical problem for prophylaxis and treatment. Therapeutics based on artemisinin combination therapies (ACTs) are currently being recommended by the World Health Organization (WHO) as the most effective medicine for the treatment of uncomplicated *P. falciparum* malaria as well as for chloroquine-resistant *P. vivax* malaria [3]. However, clinical resistance to artemisinin has been recently reported in Southeast Asia, including in the Cambodia-Thailand border region [4]. Historically, quinine and artemisinin were the main antimalarial drugs that have been used successfully against resistant malaria parasites [5, 6]. One important approach in antimalarial drug discovery and development is the investigation of potential antimalarial candidates from natural sources.

*Betula alnoides* belongs to the Betulaceae family and is mainly distributed throughout Southeast Asia and South China. In Thailand, this plant is known as Khamlang suea khrong and has been traditionally used as a tonic, stomachic, carminative, and aphrodisiac; in addition, it has been used to increase longevity and the appetite [7]. Moreover, the bark decoction of this plant has been traditionally used for the treatment of malaria infections in Northeast India [8]. Besides, betulinic acid, one of the biologically active compounds that can be found in this plant, was reported having an antiplasmodial activity against chloroquine-resistant *Plasmodium falciparum* parasites *in vitro*, with the fifty percent inhibitory concentration (IC_50_) value of 9.89 *μ*M [9].

Biological activity studies have demonstrated that methanolic and ethanolic extracts of this plant possess the following activities: anti-inflammation [10], phosphodiesterase inhibition [11], antihyperlipidaemia [12], antioxidant, antimicrobial, and *α*-glucosidase inhibition [13]. It is important to note that no scientific investigations have been carried out to substantiate the antimalarial activity of *B. alnoides*. Therefore, this study aimed at investigating the antimalarial activity of the crude aqueous stem extract of *B. alnoides* in *P. berghei*-infected mice and at evaluating the acute toxicity in mice to confirm its safety.

## 2. Materials and Methods

### 2.1. Plant Material

The stems of *B. alnoides* were collected from Chonburi Province, Eastern Thailand, in 2015 and were identified by a traditional Thai doctor, Mr. Sarupsin Thongnoppakhun. A voucher number of SKP024020101 was deposited at the Department of Pharmacognosy and Pharmaceutical Botany at the Faculty of Pharmaceutical Sciences of the Prince of Songkla University, Thailand.

### 2.2. Extraction of Plant Material

The stems of *B. alnoides* were cleaned and cut into small pieces. They were then dried in a hot air oven at 50°C for 48 h. The raw extracts were ground into coarse powder using a grinder. A total of 500 g of dried powder was extracted three times with distilled water under reflux at 60°C for 3 h. Subsequently, the extracts were filtered with Whatman no. 1 filter paper and were then concentrated to dryness with a rotary evaporator (N-1200B, EYELA) for a final extract weight of 46.21 g. The dried sample was stored in sterile, screw-capped vials at 4°C until use.

### 2.3. Experimental Animals

Animals ICR (Imprinting Control Region) mice (5–7 weeks old, weighing 20–40 g) of both sexes were used in the study. All were purchased from Nomura Siam International Co., Ltd., Pathumwan, Bangkok, Thailand. Animal experiments were carried out in accordance with the Organization for Economic Cooperation and Development (OECD) Guideline for Chemicals. The animals were housed under the following standard conditions: free access to fresh food and water, temperatures ranging from 22 to 24°C, and a standard 12 h light/dark cycle. All mice were acclimated to the environment for 1 week before the experiments began. The animal protocol was approved by the Animal Ethics Committee, Walailak University (protocol number 024/2018).

### 2.4. Four-Day Suppressive Test

The *in vivo* antimalarial activity of the aqueous stem extract of *B. alnoides* was determined using a 4-day suppressive standard test [14], which evaluated the schizonticidal activity. ICR mice were inoculated on the first day with 200 *μ*L of 1 × 10^7^ chloroquine-sensitive *P. berghei* ANKA-infected erythrocytes by intraperitoneal (i.p.) injection [15]. The malaria parasites were obtained through BEI Resources, NIAID, NIH (*P. berghei*, strain ANKA, MRA-311) and were provided by Thomas F. McCutchan. Thirty mice were then divided randomly into five groups of six mice each, with three males and three females for each group. Three groups were assigned to be the extract-treated groups. Four hours after the initiation of the infection, the mice were treated with the aqueous extract of *B. alnoides* at oral daily doses of 200, 400, and 600 mg/kg body weight for four consecutive days. The positive and negative control groups were given artesunate at oral daily doses of 6 mg/kg body weight or the vehicle (7% Tween 80 and 3% ethanol solution), respectively. In addition, the body weight of each mouse was measured before infection on day 0 and after treatment on day 4 using a sensitive digital balance (Mettler Toledo, Switzerland). On the fifth day of treatment, blood samples were collected from tail veins for thin blood smears by staining with 10% Giemsa solution. Then, each stained slide was examined under the microscope with an oil immersion objective of 100x magnification power to determine the level of parasitaemia. The percentage suppression of parasitaemia in each group of mice was calculated by comparing the mean parasitaemia in the infected control with those of the extract-treated group. Average percentage suppression was calculated using the following formula:

(1) $$\begin{array}{c} \%\text{Suppression}=\frac{\left[A-B\right]}{A}\times 100, \end{array}$$

where *A* is the average percentage of parasitaemia in the negative control group and *B* is the average percentage of parasitaemia in the extract-treated group.

### 2.5. Determination of Mean Survival Time

A total of 30 mice that were used for the 4-day suppressive test were fed *ad libitum*. The mouse mortality was observed on a daily basis until day 30 postinoculation of the parasite. Any death that occurred during this period was noted and used to determine the mean survival time during this period. The remaining mice were finally euthanized by intraperitoneal injection of a lethal dose of sodium pentobarbital (300 mg/kg body weight). The mean survival time (MST) for each group was determined by calculating the average survival time (days) of mice from the date of infection over a period of 30 days according to the following formula:

(2) $$\begin{array}{c} \text{MST}=\frac{\text{Sum of survival time of all mice in each group }\left(\text{days}\right)}{\text{Total number of mice in that group}}. \end{array}$$

### 2.6. Acute Toxicity Test

The aqueous extract of *B. alnoides* was weighed and resuspended in 7% Tween 80 and 3% ethanol solution to obtain a concentration of 5000 mg/kg body weight. Twelve ICR mice were used by randomly dividing them into two groups of six mice per group (three males and three females for each group). The mice fasted for 3 h before the experiment and were only allowed water *ad libitum*. After that, the mice in each group were fed with the extract at a single oral dose of 5000 mg/kg body weight. The control group received a single oral dose of 200 *µ*L of the vehicle (7% Tween 80 and 3% ethanol solution), which was used to dissolve the extract. Then, the general behaviour of the mice was observed continuously for 1 h, followed by intermittent observation every 4 h for a period of 24 h; thereafter, the mice were observed daily for 14 days to observe any clinical symptoms of toxicity, behavioural changes related to the central nervous, cardiovascular, and gastrointestinal systems, body weight changes, and changes in water and food consumption. At the end of the observation period, the mice were anesthetized by intraperitoneal injection of 100–150 mg/kg sodium pentobarbital. Blood samples were collected by cardiac puncture and were used to measure the biochemical parameters. Subsequently, the abdominal cavity was opened and the livers, kidneys, and spleens were immediately removed from all animals for histopathological examination.

### 2.7. Biochemical Analysis

At the end of the oral toxicity assays, the biochemical parameters of liver function tests (total protein, albumin, alanine aminotransferase (ALT), aspartate aminotransferase (AST), and alkaline phosphatase (ALP)) and kidney function tests (blood urea nitrogen (BUN) and creatinine) were determined by standard techniques in an AU480 chemistry analyser (Beckman Coulter, Brea, CA, USA).

### 2.8. Histopathological Examination

Tissue samples of the liver, kidney, and spleen were processed in the Department of Tropical Pathology of the Faculty of Tropical Medicine, Mahidol University, Thailand. Briefly, all tissue samples were fixed in 10% formalin at room temperature for 24–48 h. Following fixation, the tissues were dehydrated in ascending grades of ethanol followed by xylene and were embedded in paraffin blocks. The paraffin blocks of each tissue sample were sectioned to a thickness of 5 *μ*m and were stained with Harris' haematoxylin and eosin. The stained sections were dehydrated by increasing the concentrations of ethanol; this was followed by a xylene wash, and the sections were mounted with glass coverslips. To evaluate the histopathological changes, the stained slides were observed under light microscopy by two independent observers who were blinded to the experimental groups.

### 2.9. Statistical Analysis

All quantitative data are expressed as the mean ± SEM (standard error of means). Statistical analysis was carried out using SPSS statistical software version 21 (SPSS, IL, USA). The normality of distribution was tested using the Kolmogorov–Smirnov test. The statistical significance of the mean parasitaemia suppression, body weight, and survival time differences between the groups was computed by one-way ANOVA followed by post hoc Tukey's multiple comparison test. A *P* value of less than 0.05 was considered statistically significant.

## 3. Results

### 3.1. Four-Day Suppressive Effect

The aqueous stem extract of *B. alnoides* decreased the level of parasitaemia and significantly suppressed the parasites in *P. berghei*-infected mice in a dose-dependent manner compared to the suppressive effects in the negative control mice. As shown in Table 1, the treatments of the mice with daily doses of 200, 400, and 600 mg/kg body weight of the extract exhibited high antimalarial activities with 46.90, 58.39, and 71.26% suppression, respectively. The comparison analysis revealed that all dosages of the extract showed statistically significant differences (*p* < 0.05) on the 5^th^ day of parasitaemia compared to that of the negative control. The standard drug artesunate has a potent suppression of 96.12% at the dose of 6 mg/kg body weight, which was higher than those of the extract-treated groups. In addition, the survival time of all animals was assessed over a period of 30 days, as shown in Table 1. Nontreated mice began to die on the 5^th^ day after infection, and the mean survival time was 5.5 days; mice treated with artesunate started to die on the 8^th^ day after infection and had an average survival time of 9.0 days. For the extract-treated groups, the mean survival time was dose-dependent. Mice treated with the extract at doses of 200, 400, and 600 mg/kg body weight remained alive for 8.5, 15.0, and 22.5 days, respectively. However, only extract doses of 400 and 600 mg/kg body weight significantly prolonged the survival time compared to the survival time of the negative control mice (*p* < 0.05). It is important to note that the extract exhibited a signiﬁcant improvement in the survival time of mice that was higher than the survival time of mice treated with artesunate, the positive control.

### 3.2. Effect of Extract on Body Weight

The body weight changes were observed between days 0 and 4 in all groups of mice. There was the highest degree of weight reduction from day 0 to day 4 in the negative control group (−16.25% ± 0.49%); however; this weight reduction was not statistically significant. Treatment with the aqueous stem extract of *B. alnoides* significantly prevented the loss of body weight on day 4 compared to the body weights on day 0 at all dose levels. The results also revealed that the extract-treated groups at all dose levels significantly prevented the loss of body weight compared to that of the negative control. On day 4, the percent change in body weight increased in a dose-dependent manner (Table 2). When compared amongst themselves, an extract dose of 600 mg/kg body weight showed a statistically significant (*p* < 0.05) protection against weight loss compared to the protection of a dose of 200 mg/kg body weight. However, no significant difference was observed with a dose of 400 mg/kg body weight. Remarkably, the extract at the dose of 600 mg/kg body weight prevented a reduction of the body weights of the infected mice compared with that of the artesunate-treated group.

### 3.3. Acute Toxicity Study

#### 3.3.1. Effects of *B. alnoides* Extract on General Health and Behaviour

The results revealed that all male and female mice in the extract-treated group exhibited no gross physical and behavioural changes, such as vomiting, ataxia, excitement, sleep, diarrhoea, abnormal secretion, or hair erection; in addition, all mice survived during the 14-day observation period. It is important to note that altered feeding was observed, and the food and water intake increased more than normal.

#### 3.3.2. Effects of *B. alnoides* Extract on Food and Water Consumption and Body Weight

Food and water consumption by the *B. alnoides* extract-treated mice at the dose of 5000 mg/kg body weight significantly increased during the first 3 days of the observation period compared to the consumption of the negative control group. After this, the increased water and food intake completely disappeared, and all animals in the treated group became normal; there were no differences between the groups (Table 3). The effect of *B. alnoides* extract on the body weight of each mouse was recorded before the start of the treatment with the extract (day 0) and at the end of the experimental period (day 14). The mean body weight of the *B. alnoides* extract-treated mice at the dose of 5000 mg/kg body weight (33.78 ± 2.16 g) significantly increased when compared with the mean body weight of the negative control mice (31.74 ± 1.37 g).

#### 3.3.3. Effects of *B. alnoides* Extract on Biochemical Parameters

The effects of acute oral treatment with 5000 mg/kg aqueous extract of *B. alnoides* on the biochemical parameters are shown in Table 4. The total levels of protein, albumin, and liver enzymes, including aspartate transaminase (AST), alanine aminotransferase (ALT), and alkaline phosphatase (ALP), from mice treated with 5000 mg/kg body weight of extract were not significantly altered compared to the levels of the negative control mice. The BUN and creatinine levels in mice treated with 5000 mg/kg body weight of extract were not significantly different compared to the levels in the negative control mice. In addition, all biochemical parameters for the liver and kidney function tests were not significantly different between the treated and untreated groups for both the male and female mice.

#### 3.3.4. Effects of *B. alnoides* Extract on the Histopathology of the Liver, Kidneys, and Spleen

**Figure 1:**
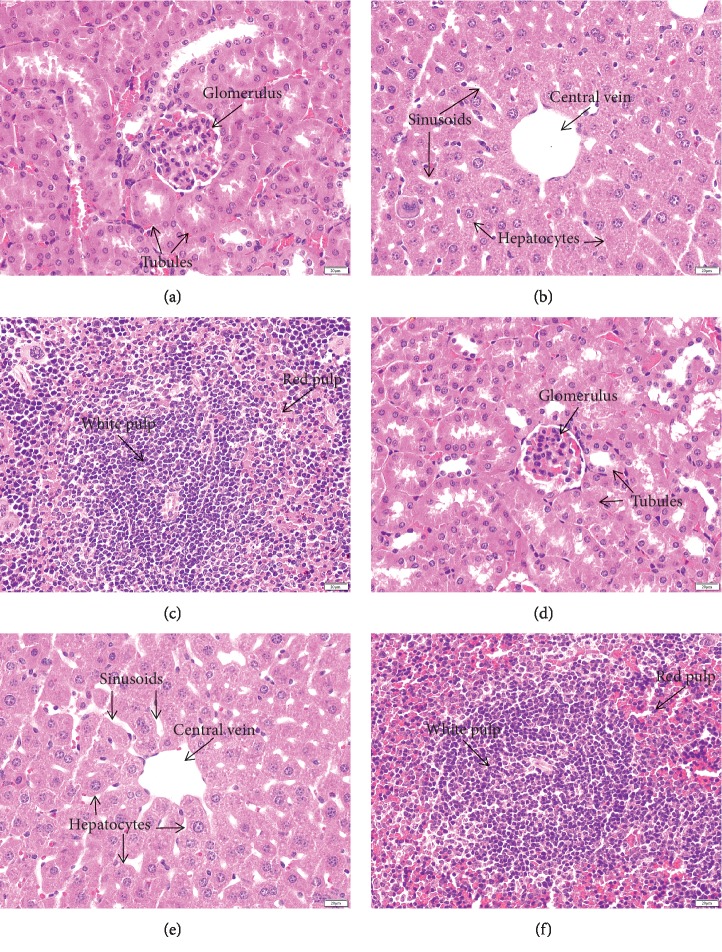
Histopathological examination of the kidneys, liver, and spleen. The kidneys, liver, and spleen from the negative control group (a, b, c) and from the group treated with 5000 mg/kg of the aqueous extract of *B. alnoides* (d, e, f). All images are 400x magnification. Bars = 20 *μ*m.

The histological examinations of liver, kidney, and spleen sections from mice treated with 5000 mg/kg body weight of extract showed normal morphology and histology compared to those of the negative control mice (Figure 1). The results from the oral toxicity evaluation indicated that there was virtually no toxicity caused by the extract at the maximum single oral dose of 5000 mg/kg body weight (acute toxicity). In addition, there were no differences in the histopathological examinations of the liver, kidneys, and spleen between the male and female mice in the treated group.

## 4. Discussion

In this study, chloroquine-sensitive *P. berghei* was used for the induction of malaria because of its ability to produce a rodent model of malaria that is similar to human malaria infection [16]. To determine the *in vivo* antimalarial activity, the 4-day suppressive model has been widely used and accepted as a viable model to evaluate the effects of candidate agents on early malaria infections [17, 18]. The current study shows that the stem extract of *B. alnoides* significantly decreased the level of parasitaemia in *P. berghei*-infected mice and extract dose of 600 mg/kg body weight prolonged the survival time of the mice up to 22.5 days. These findings are in agreement with a previous study that demonstrated the parasitaemia suppression of more than 30% and prolonged survival time of the treated mice compared with those of the negative control group; these parameters are often considered to be signs of effective treatment in standard screening tests [18].

Body weight loss is one of the general symptoms of rodent and human malarial infections. A potent antimalarial is expected to prevent body weight loss in infected mice that have increasing levels of parasitaemia [16]. In the 4-day suppressive test, the body weight of each infected mouse was measured before the infection on day 0 and after treatment on day 4. Interestingly, the stem extract of *B. alnoides* at all dose levels could preserve the body weight loss of infected mice. The results correspond to the increase in food and water consumption, which was observed in mice treated with the extract at a dose of 5000 mg/kg body weight for the acute toxicity study. This protective effect of the extract on the body weight loss that is associated with malaria infections might be related to the appetizing property of this plant, which is traditionally used in Thailand for the treatment of the loss of appetite.

The levels of BUN and creatinine are biomarkers for the assessment of kidney function. The increased blood content of creatinine has been reported in renal injury; this is the same as the urea level, which is increased in conditions with kidney damage and in conditions with improperly functioning kidneys [19]. In this study, the detection of these parameters was performed, and the results showed that the BUN and creatinine levels of the extract-treated group were not significantly different compared to the levels in the negative control, indicating that the extract at the dose of 5000 mg/kg body weight has no nephrotoxic action. The liver is the major organ affected by toxicity. The injury of the liver may affect the integrity of hepatocytes and may lead to the release of membrane-bound enzymes (e.g., ALT and AST). In addition, damage to the hepatobiliary system causes the release of essential enzymes (e.g., ALP) into the bloodstream. The alteration of these enzyme levels is used as a diagnostic measure of liver damage [20, 21]. This study clearly indicated that the liver enzymes and the other biochemical parameters of the liver function test were not significantly altered compared to those of the negative control. Therefore, the results from the oral toxicity evaluation indicated that the aqueous stem extract of *B. alnoides* did not exert toxic effects since there was no alteration in the biochemical parameters and no effect on the structures of the kidneys and liver, and none of the extracts caused mortality, even in concentrations of up to 5000 mg/kg body weight. These results support the safe use of this plant in traditional medicine.

*In vivo* antimalarial activity can be classified as moderate, good, or very good if an extract displays a percentage of parasite suppression equal to or greater than 50% at doses of 500, 250, and 100 mg/kg body weight per day [22]. Based on this classification, the present study clearly showed that the extract could reduce the level of parasitaemia with percent suppressions of 58.39 and 71.26 at doses of 400 and 600 mg/kg body weight, respectively. Accordingly, the aqueous stem extract of *B. alnoides* exhibited good antimalarial activity. Artesunate, a standard drug used for positive control, reduced the level of parasitaemia with a high suppression (96.13%). These results are in agreement with the results of other studies that show a suppression of 98.82% at a dose of 5 mg/kg body weight [23, 24]. The lower suppression of extract-treated groups compared to the suppression by artesunate treatment may be due to the limitation of several parameters, including poor bioavailability and the slow absorption of the active compound in the extract.

Some secondary metabolites of plants, such as alkaloids, flavonoids, lignans, and terpenoids, have been reported to possess antiparasitic properties that can inhibit the proliferation of malaria parasites *in vitro* and can control infections of *P. berghei* in mice [24]. Since then, triterpenoid compounds, such as taraxerone, lupeol, betulin, betulinic acid, oleanolic acid, and ursolic acid, have been reported to be found predominantly in the stem bark and twigs of *B. alnoides* [25]. Therefore, triterpenoids may be the active components that contribute to the antimalarial activity of *B. alnoides* extract.

## 5. Conclusions

For the first time, this study reports the *in vivo* antimalarial activity of *B. alnoides* extract. The aqueous stem extract possesses good antimalarial activity. The extract at a single oral dose of 5000 mg/kg body weight had no serious toxic effects on the biochemical parameters or the histopathological appearance of the livers, kidneys, and spleens of mice. The results of this study provide a basis for further investigation to determine the antimalarial mechanism of action by *B. alnoides* and to investigate the active compounds of this plant with the purpose of discovering candidate compounds for the development of novel antimalarial drugs.

## Figures and Tables

**Table 1:** Four-day suppressive activity of the aqueous stem extract of *B. alnoides* compared with the suppressive activities of artesunate and the negative control against *P. berghei*-infected mice.

Groups	% parasitaemia	% suppression	MST (days)
Negative control	15.96 ± 0.06	—	5.5 ± 0.50
Artesunate 6 mg/kg	0.62 ± 0.38^a,b,c^	96.13 ± 2.38	9.0 ± 1.00
Extract 200 mg/kg	8.50 ± 0.17^a^	46.90 ± 1.07	8.5 ± 0.50
Extract 400 mg/kg	6.66 ± 0.18^a,b^	58.39 ± 1.15	15.0 ± 1.30^d^
Extract 600 mg/kg	4.60 ± 0.22^a,b,c^	71.26 ± 1.40	22.5 ± 1.19^d,e^

Data are expressed as the mean ± SEM (*n* = 6/group), MST: mean survival time. Values are significantly different at *p* < 0.05. ^a^Significantly lower than that of the negative control. ^b^Significantly lower than those of the groups treated with 200 mg/kg extract. ^c^Significantly lower than those of the groups treated with 400 mg/kg extract. ^d^Significantly longer than those of the negative control and the groups treated with artesunate and 200 mg/kg extract. ^e^Significantly longer than those of the negative control and the groups treated with 400 mg/kg extract.

**Table 2:** Effect of aqueous stem extract of *B. alnoides* on the body weights of infected mice in a 4-day suppressive test.

Groups	Mean body weight (g)		% change
	Day 0	Day 4	
Negative control	29.80 ± 2.12	24.95 ± 1.67	−16.25 ± 0.49
Artesunate 6 mg/kg	31.60 ± 1.35	30.45 ± 0.89	−3.57 ± 1.82^b^
Extract 200 mg/kg	28.89 ± 1.83	25.55 ± 1.48^a^	−11.47 ± 0.91^b,c^
Extract 400 mg/kg	28.99 ± 0.81	26.49 ± 0.77^a^	−8.65 ± 0.68^b,c^
Extract 600 mg/kg	28.37 ± 0.99	26.16 ± 0.97^a^	−7.80 ± 0.30^b,d^

Data are expressed as the mean ± SEM (*n* = 6/group), day 0: before treatment; day 4: after completing treatment. ^a^Significantly higher than that of the negative control. ^b^Significantly higher than that of the negative control. ^c^Significantly higher than those of the groups treated with artesunate. ^d^Significantly higher than those of the groups treated with 200 mg/kg extract.

**Table 3:** Evaluation of food and water consumption by both the negative control group and the group treated with the aqueous stem extract of *B. alnoides* at the dose of 5000 mg/kg.

Parameters	Negative control	5000 mg/kg extract
Food consumption (g)		
Week 1: days 1–3	20.30 ± 0.67	37.67 ± 1.17^a,1^
Week 1: days 4–7	19.00 ± 0.41	20.25 ± 0.63
Week 2: days 8–14	19.29 ± 0.41	19.71 ± 0.46

Water consumption (mL)		
Week 1: days 1–3	19.67 ± 0.33	37.00 ± 2.00^b,2^
Week 1: days 4–7	20.25 ± 0.63	21.75 ± 0.63
Week 3: days 8–14	18.43 ± 0.43	28.86 ± 0.47

Data are expressed as the mean ± SEM (*n* = 6/group). ^a^Significantly higher food consumption than that of negative control. ^1^Significantly higher food consumption than those of the groups treated with 5000 mg/kg extract on days 4–7 and days 8–14. ^b^Significantly higher water consumption than that of the negative control. ^2^Significantly higher water consumption than those of the groups treated with 5000 mg/kg extract on days 4–7 and days 8–14.

**Table 4:** Biochemical parameters of the negative control group and the group treated with the aqueous extract of *B. alnoides*.

Parameters	Negative control		5000 mg/kg	
	Male	Female	Male	Female
Liver function tests				
Total protein (g/L)	4.30 ± 0.10	4.63 ± 0.13	4.47 ± 0.23	4.73 ± 0.07
Albumin (g/L)	2.69 ± 0.01	2.70 ± 0.00	2.69 ± 0.01	2.70 ± 0.10
AST (U/L)	138.87 ± 1.73	141.00 ± 1.00	137.28 ± 3.58	135.32 ± 2.54
ALT (U/L)	85.27 ± 0.17	85.60 ± 0.35	85.60 ± 0.98	85.43 ± 3.08
ALP (U/L)	81.13 ± 1.11	79.37 ± 0.41	80.13 ± 1.76	79.38 ± 0.51

Kidney function tests				
BUN (mg/dL)	26.21 ± 0.97	26.00 ± 0.00	27.57 ± 1.58	23.50 ± 1.75
Creatinine (mg/dL)	0.13 ± 0.02	0.21 ± 0.03	0.14 ± 0.01	0.18 ± 0.02

## Data Availability

The data used to support the findings of this study are included within the article.
